# P-844. Piloting Post Prescriptive Audit and Feedback for Outpatient Antimicrobial Prescriptions in a Tertiary Care Centre from Resource Limited Setting Effectively Reduced Quinolone Prescriptions for Respiratory Infections: Results from a Quasi experimental Study

**DOI:** 10.1093/ofid/ofaf695.1052

**Published:** 2026-01-11

**Authors:** Merlin Moni, Akita Ajay, Dipu T Sathyapalan, Swathy S Samban, Geetha M, Boddu Madan Gopal, Lokesh Yarramallu, J S Gayathri, Ananth Ram K J

**Affiliations:** Amrita Institute of Medical Sciences, Kochi, Kochi, Kerala, India; Research Assistant, Clinical Pharmacist, Infectious diseases department, Amrita Institute of Medical Sciences, Kochi, Kerala, India; Professor, Department of Internal Medicine, Lead Division of Infectious diseases, Administrative chair, URUM, chairman HICC, Kochi, Kerala, India; Amrita Institute of Medical Sciences and Research Centre, Kazhkkuttom, Kerala, India; Amrita Vishwa Vidyapeetham, Kollam, Kerala, India; Amrita School of Computing, Parvathipuram, Andhra Pradesh, India; Amrita Vishwa Vidhyapeetham, rajahmundry, Andhra Pradesh, India; Amrita Institute of Medical Sciences, Kochi, Kerala, India; Clinical Pharmacist, Amrita Institute of Medical Sciences,AMRITA VISHWA VIDYAPEETHAM, Kochi, Kerala, India

## Abstract

**Background:**

Unchecked outpatient (OP) antibiotic use significantly contributes to the global challenge of AMR in LMICs, where stewardship efforts are mostly inpatient-focused. Outpatient prescriptions account for up to 95% of antibiotic use. Despite existing inpatient programs, outpatient stewardship frameworks are underdeveloped in LMICs. This study evaluated the feasibility and impact of implementing outpatient stewardship in a tertiary hospital in South India.

**Figure:**
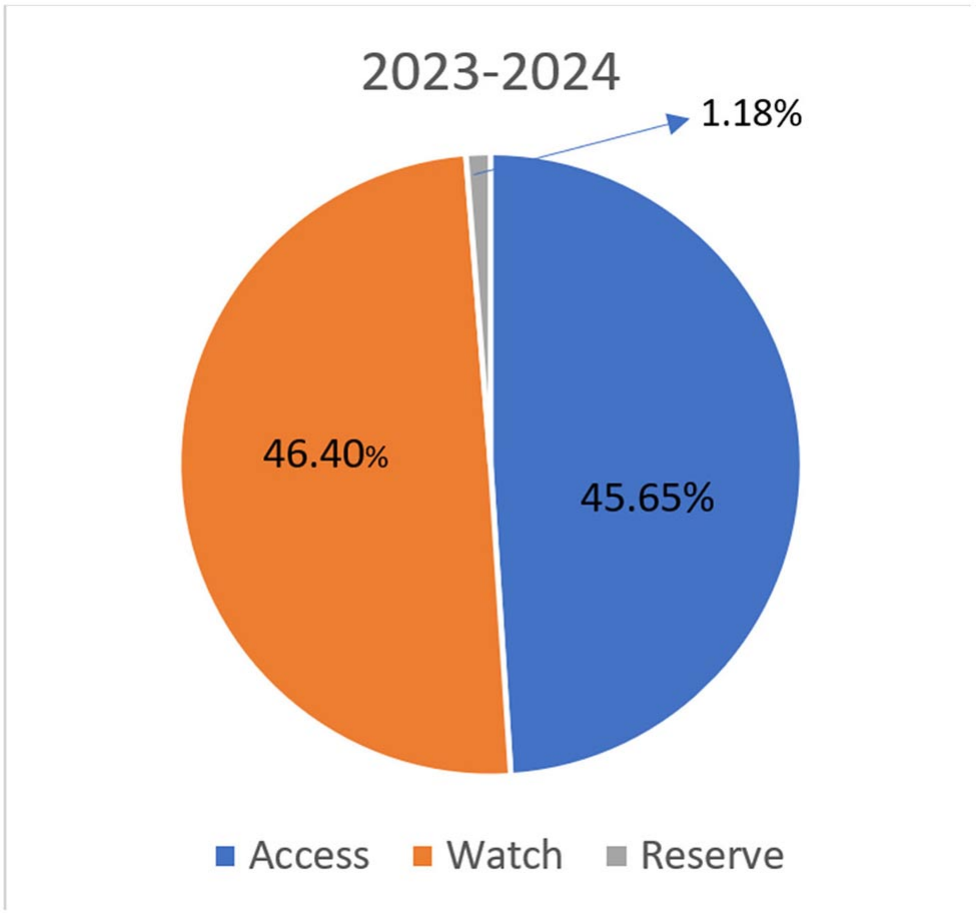
Distribution of Antibiotic Prescriptions by AWaRe Classification

**Figure:**
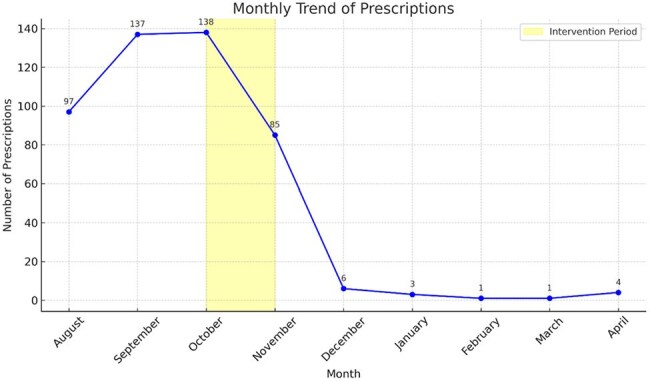
Monthly Levofloxacin Prescription Trends in Outpatient Settings in a Tertiary Care Centre

**Methods:**

Study Setting: Tertiary academic centre in South India with established Inpatient antimicrobial stewardship.

Study Design: Quasi-experimental pre-post study.

Baseline Audit: A retrospective review of 2023–2024 data yielded n=47,673 antibiotic prescriptions from outpatient department. AWaRe classifications are shown in Figure 1. In 2023–2024, n=46.4% (n=the number that represent the percentage) of OP prescriptions were Watch antibiotics, with high levofloxacin use (n=3,142). Interventions was planned to reduce Watch group use, with special focus on levofloxacin for respiratory infections, and prioritize Access antibiotics.

Intervention Phase (Oct–Nov 2024): Included WHO AWaRe sensitization via educational materials, tent cards, and a video(figure 4). IT-based interventions included mandatory indication fields and AWaRe color-coded alerts in the electronic health record. A peer comparison tool generated monthly feedback on outpatient levofloxacin use (figure 3).

Monthly Audit: Tracking monthly levofloxacin prescriptions and feedback respectively.

**Figure:**
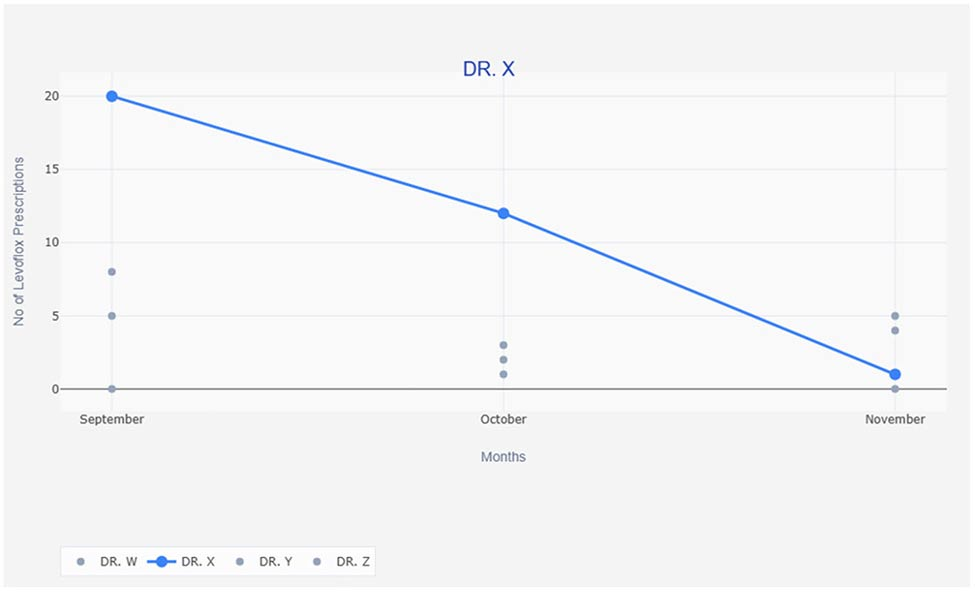
Peer Comparison Tool: Visual Snapshot

**Figure:**
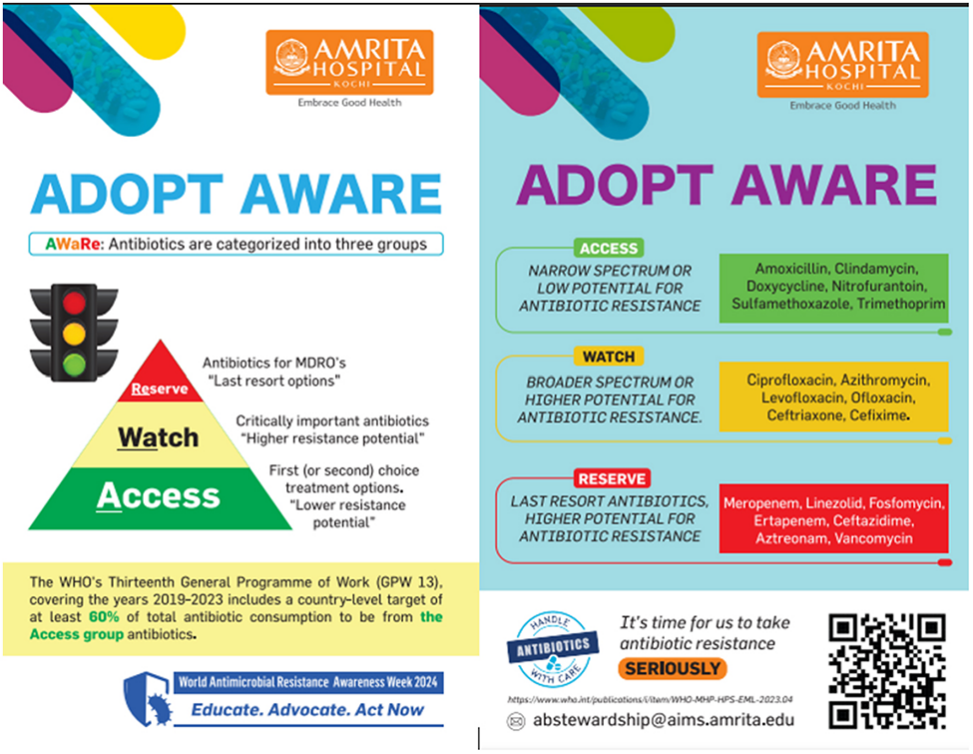
Tent Cards deployed in Outpatient Settings Tent cards on AWaRe classification was deployed in the hospital Outpatient clinics.

**Results:**

Around 95% reduction in levofloxacin prescribing post-intervention (figure 2).

**Conclusion:**

Increased AWaRe awareness, improved Access antibiotic use, and successful outpatient stewardship integration were observed. Implementing post prescriptive audit and IT based feedback for outpatient antimicrobial prescriptions proved feasible and effectively reduced quinolone prescriptions for respiratory infections.

**Disclosures:**

All Authors: No reported disclosures

